# Voltammetric determination of vitamin B6 in the presence of vitamin C based on zinc ferrite nano-particles modified screen-printed graphite electrode

**DOI:** 10.5599/admet.1702

**Published:** 2023-03-15

**Authors:** Peyman Mohammadzadeh Jahani, Maedeh Jafari, Sayed Ali Ahmadi

**Affiliations:** 1 School of Medicine, Bam University of Medical Sciences, Bam, Iran; 2 Department of Pediatrics, School of Medicine, Kerman University of Medical Sciences, Kerman, Iran; 3 Department of Chemistry, Kerman Branch, Islamic Azad University, Kerman, Iran

**Keywords:** Zinc ferrite nanoparticles, voltammetry, vitamin B6, vitamin C, modified electrode

## Abstract

The zinc ferrite nano-particles (ZnFe_2_O_4_) modified screen-printed graphite electrode (ZnFe_2_O_4_/SPGE) was used for the voltammetric determination of vitamin B_6_ in real samples, using differential pulse voltammetry (DPV). It has been found that the oxidation of vitamin B_6_ at the surface of such an electrode occurs at a potential about 150 mV less positive compared to an unmodified screen-printed graphite electrode. After optimization, a vitamin B6 sensor with a linear range from 0.8 to 585.0 μM and a detection limit of 0.17 μM. The ZnFe_2_O_4_/SPGE sensor exhibits good resolution between the voltammetric peaks of vitamin B_6_ and vitamin C, making it suitable for detecting vitamin B_6_ in the presence of vitamin C in real samples.

## Introduction

Vitamins are small organic molecules whose lack or excess may result in several diseases to the organisms that need them. They are classified into two groups by their solubilities, namely water-soluble vitamins (vitamin B and vitamin C) and fat-soluble vitamins (vitamin A, vitamin D, vitamin E, and vitamin K) [1 2].

Vitamin B_6_ belongs to the water-soluble B complex vitamins group, commonly called pyridoxine. It is essential in the diet for the metabolism of amino acids and the maintenance of body cells. The nervous and immune systems need vitamin B_6_ for efficient functioning; it also plays a major role in the conversion of tryptophan to niacin [3,4]. Also, It is found in different chemical forms (pyridoxamine, pyridoxine or 5-phosphate derivatives), but the most stable form is pyridoxine which is used in drug formulations such as multivitamin supplements or in enriched foods [5].

Vitamin C is one of the most important water-soluble vitamins and it refers to all compounds exhibiting equivalent biological activity to L-ascorbic acid (AA), including dehydroascorbic acid (DHAA), the oxidation product of AA, its isomers and esters. Vitamin C is an antioxidant necessary for the growth, development, and repair of all tissues [6,7]. Low levels of vitamin C can result in a condition called scurvy. Scurvy may cause symptoms such as rash, muscle weakness, joint pain, tiredness, or tooth loss. Vitamin C is an important antioxidant, along with vitamin E, beta-carotene, and many other plant-based nutrients. Antioxidants block some of the damage caused by free radicals, substances that damage DNA [8,9].

Studies show that vitamins B_6_ and C are essential for the natural synthesis of dopamine in the human body. On the other hand, large doses of vitamins B_6_ and C may reduce the risk of kidney stone formation in women. So, the simultaneous determination of these compounds is very important for pharmaceutical and biological investigation [10,11].

Several methods have been developed to determine vitamins B_6_ and C, including high-performance liquid chromatography [12], spectrophotometry [13], and flow injection [14,15]. However, these methods require not only advanced technical expertise but also time-consuming, expensive and often need the pretreatment step. Electrochemical detection is an attractive alternative approach to these technologies because of the inherent advantages of simplicity, ease of miniaturization, cost-effectiveness, dependability, high sensitivity, and relatively low cost [16-21]. So far, electrochemical sensors have found widespread use in a variety of disciplines, including pharmaceutical, food, and clinical analyses [22-29].

Currently, the advancement of screen-printing technology for the fabrication of screen-printed electrodes (SPEs) is attracting enormous attention due to the advantageous properties of SPEs compared to conventional electrodes, such as cost-effectiveness, disposability, simplicity, versatility, availability of materials and patterns, elimination of electrode maintenance, the requirement for low volumes of solution, and appropriateness for outside laboratory measurement [30-32]. Chemically modified electrodes are best suited for the electrochemical determination of pharmaceutical, environmental, or biological samples. Chemically modified electrodes reduce the over-potential required for either the oxidation or reduction of the electro-active compounds [33-39]. Also, modification of electrodes is a powerful strategy for overcoming such limitations of un-modified electrodes as low selectivity, poor sensitivity, low stability, and the blockage of the electron transfer [40-45].

Nanostructured metal oxides crystallizing in the spinel structure type have been investigated intensively over the years and present a permanent interest due to their wide technological applications such as magnetic and optical materials, semiconductors, pigments, catalysts, or material for biomedical applications [46-51].

Ferrites are a well-known class of complex oxides of considerable technological importance. On the other side, nano ZnFe_2_O_4_ as spinel ferrites is found to be one of the most interesting spinel systems because of its unique properties, photochemical stability, good visible-light response and favourable magnetism [52,53]. A characteristic of ZnFe_2_O_4_ is that it has two different metal cations, Zn and Fe, with O as an anion. The cations occupy two different positions in a spinel structure: tetrahedral (Zn) and octahedral (Fe) sites along the face-centered cubic lattice formed by O_2_^-^ cations. The use of bimetallic oxides as electrode materials could enhance both electrical conductivity by two orders of magnitude and electrochemical activity versus materials prepared with unitary metal oxides [54,55].

In the present work, the preparation and application of a screen-printed graphite electrode, modified with zinc ferrite nano-particles (ZnFe_2_O_4_), for the determination of vitamin B_6_ in the presence of vitamin C is described. The electrochemical behavior of vitamin B_6_ at ZnFe_2_O_4_/SPGE was investigated. The results showed the superiority of ZnFe_2_O_4_/SPGE to the bare electrode in terms of better sensitivity. We have also evaluated the analytical performance of the ZnFe_2_O_4_/SPGE for the quantification of vitamin B_6_ in the presence of vitamin C in some real samples.

## Experimental

### Chemicals and instrumentation

All chemicals used were of analytical reagent grade purchased from Sigma-Aldrich and were used as received without any further purification. Double-distilled water was used throughout all experiments. Orthophosphoric acid was utilized to prepare the phosphate buffer solutions (PBSs), and sodium hydroxide was used to adjust the desired pH values (pH range between 2.0 and 9.0).

Cyclic voltammetry (CV), linear sweep voltammetry (LSV), chronoamperometry, and differential pulse voltammetry (DPV) investigations were performed in an electroanalytical system Autolab PGSTAT302N, potentiostat/galvanostat connected to an electrode cell, the SPGE (DropSens; DRP-110: Spain), containing graphite counter electrode, a graphite working electrode, and a silver pseudo-reference electrode. The system was run on a PC using General Purpose Electrochemical System (GPES) software. Solution pH values were determined using a 713 pH meter combined with a glass electrode (Metrohm, Switzerland).

### Preparation of modified electrode

ZnFe_2_O_4_ nano-particles were used to coat the bare screen printed graphite electrode. A stock solution of ZnFe_2_O_4_ nano-particles in 1 mL of the aqueous solution was prepared by distributing 1 mg of ZnFe_2_O_4_ nano-particles via ultra-sonication for 50 min, whereas 4 μL of aliquots of the ZnFe_2_O_4_ nano-particles suspension solution was cast on carbon working electrodes and evaporated at room temperature.

## Results and discussion

### Electrochemical behavior of vitamin B6 on the ZnFe2O4/SPGE

**Scheme 1.**
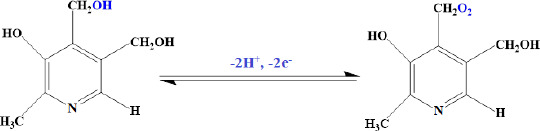
The proposed mechanism for the oxidation of vitamin B_6_ at the ZnFe_2_O_4_/SPGE.

According to our knowledge, the electrooxidation of vitamin B_6_ depends on the pH value of the solution (Scheme 1). So, the effect of pH was investigated using the DPV method. Results show that the oxidation peak current increased slowly from pH 2.0 to 7.0, and then the current conversely decreased when the pH value increased from 7.0 to 9.0. Consequently, pH 7.0 was chosen as the optimal experimental condition for other experiments.

**Figure 1.**
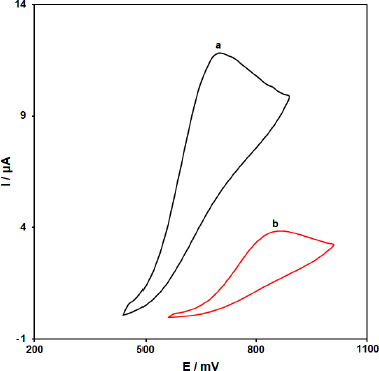
Cyclic voltammograms of a) ZnFe_2_O_4_/SPGE and b) SPGE in the presence of 200.0 μM vitamin B_6_ at a pH 7.0 of *0.1 M PBS*, respectively.

To investigate the vitamin B_6_ behavior and the as-produced electrode response to vitamin B_6_, the performance of ZnFe_2_O_4_/SPGE was compared to that of unmodified SPGE. Figure 1 shows the CV curve obtained for ZnFe_2_O_4_/SPGE (curve a) and unmodified SPGE (curve b) in the presence of 200.0 μM vitamin B_6_-containing PBS at the scan rate of 50 mV/s. The results showed that the oxidation of vitamin B_6_ is very weak on the surface of the bare SPGE, but the presence of ZnFe_2_O_4_ nano-particles could enhance the peak current and decrease the oxidation potential (decreasing the overpotential). A substantial negative shift of the currents starting from oxidation potential for vitamin B_6_ and a dramatic increase of the current indicates the catalytic ability of ZnFe_2_O_4_/SPGE to vitamin B_6_ oxidation. The results showed that the use of ZnFe_2_O_4_ nano-particle improved the characteristics of vitamin B_6_ oxidation, which was partly due to excellent characteristics of ZnFe_2_O_4_ nano-particles such as excellent electrical conductivity and good chemical stability.

### Effect of scan rate

**Figure 2.**
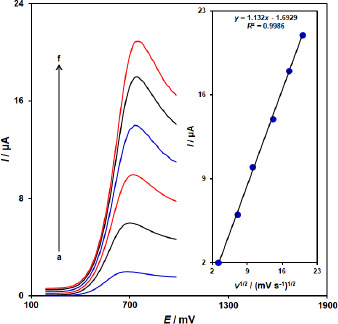
Linear sweep voltammograms of vitamin B_6_ (100.0μM) at ZnFe_2_O_4_/SPGE at different scan rates of a) 10, b) 50, c) 100, d)200, e) 300, and f) 400 mV/s in 0.1 M PBS (pH 7.0). Insert: Plot of *I*p versus *ν* ^1/2^ for the oxidation of vitamin B_6_ at ZnFe_2_O_4_/SPGE.

The linear sweep voltammograms measurements were carried out to evaluate the association of peak current with scan rate at varied scan rates (10-400 mV/s) in the 100.0 μM vitamin B_6_-containing 0.1 M PBS (pH = 7.0) on the ZnFe_2_O_4_/SPGE (Figure 2). As shown in Figure 2, the peak currents of vitamin B_6_ grow with increasing scan rates and there are good linear relationships between the peak currents (*I*_p_) and the square root of the scan rate (*ν* ^1/2^). The results also showed that the action is mass transfer of vitamin B_6_ controlled at diffusion process.

**Figure 3.**
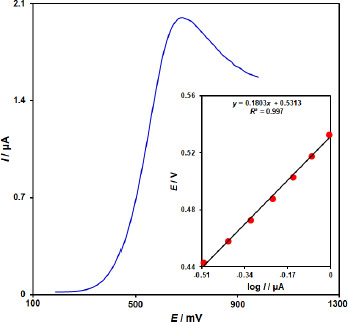
Linear sweep voltammogram for ZnFe_2_O_4_/SPGE in the presence of 0.1 M PBS (Ph 7.0) with 100.0 μM of vitamin B_6_ at the scan rate of 10 mV/s; Points: outputs used in Tafel plot; Inset: Tafel plot of LSV.

To obtain further information on the rate-determining step, a Tafel plot was developed for the vitamin B_6_ at the surface of ZnFe_2_O_4_/SPGE using the data derived from the rising part of the current–voltage curve (Figure 3). The slope of the Tafel plot is equal to 2.3RT/n(1 - α)F, which comes up to 0.1803 V decade^-1^. We obtained the charge transfer coefficient (α) as 0.67.

### Chronoamperometric measurements

**Figure 4.**
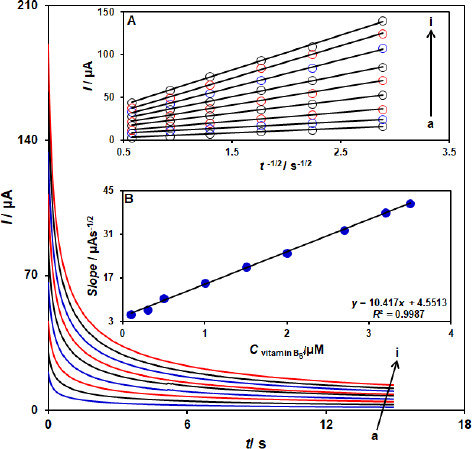
Chronoamperograms obtained at the ZnFe_2_O_4_/SPGE in the presence of a) 0.1, b) 0.3, c) 0.5, d) 1.0, e) 1.5, f) 2.0, g) 2.7, h) 3.2, and i) 3.5 μM vitamin B_6_ in the0.1 M buffer solution (pH 7.0). A) Plot of *I* versus *t*^-1/2^ for electrooxidation of vitamin B_6_ obtained from chronoamperograms a–i. B**)** Plot of slope from straight lines versus vitamin B_6_ level.

The electrooxidation of vitamin B_6_ by a ZnFe_2_O_4_/SPGE was also studied by chronoamperometry (Figure 4). Chronoamperometric measurements of different concentrations of vitamin B_6_ at the ZnFe_2_O_4_/SPGE sensor were accomplished by setting the working electrode potential at 760 mV as the first step potential. Using chronoamperometric studies, we determined the diffusion coefficient, *D*, of vitamin B_6_ in a buffer solution. The experimental plots of *I*_p_ versus *t*^-1/2^ were employed with the best fits for different concentrations of vitamin B_6_ (Figure 4A). The slopes of the resulting straight lines were then plotted versus vitamin B_6_ concentrations (Figure 4B). Using these slopes and the Cottrell equation, we obtained *D*= 9.1×10^-6^ cm^2^ s^-1^.

### Calibration plot and limit of detection

**Figure 5.**
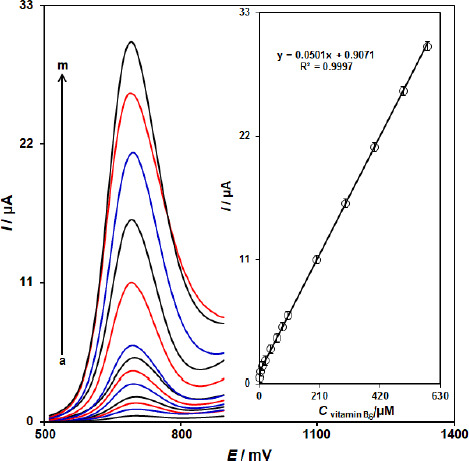
DPV curves of ZnFe_2_O_4_/SPGE in the 0.1 M buffer solution (pH 7.0) containing different concentrations of vitamin B_6_. a-m corresponds to 0.8, 5.0, 10.0, 20.0, 40.0, 60.0, 80.0, 100.0, 200.0, 300.0, 400.0, 500.0, and 585.0 μM vitamin B_6_. Inset: Plots of electrocatalytic peak current as a function of vitamin B_6_ concentration.

Since DPV has a much higher current sensitivity and better resolution than CV and LSV, DPV was used for the determination of vitamin B_6_. Figure 5 shows the DPV curves of ZnFe_2_O_4_/SPGE in the PBS buffer with variable vitamin B_6_ levels (Step potential=0.01 V and pulse amplitude=0.025 V). It was found that the electrocatalytic peak currents of vitamin B_6_ oxidation at ZnFe_2_O_4_/SPGE surface linearly depended on vitamin B_6_ concentrations above the range of 0.8-585.0 μM (with a correlation coefficient of 0.9997), while determination limit was achieved to be 0.17 μM.

### Determination of vitamin B6 in the presence of vitamin C

**Figure 6.**
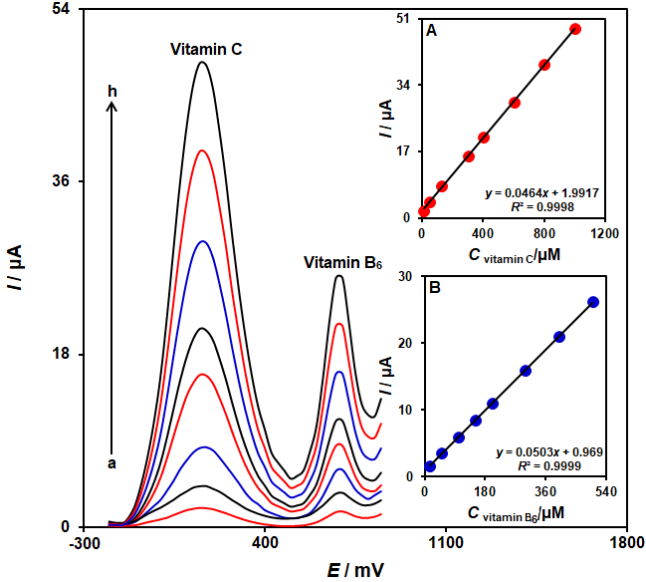
differential pulse voltammograms of ZnFe_2_O_4_/SPGE in 0.1 M PBS (pH 7.0) containing different concentrations of vitamin C and vitamin B_6_ mixed solutions of: a)10.0+15.0, b) 50.0+50.0, c) 125.0+100.0, d) 300.0+150.0, e) 400.0+200.0, f) 600.0+300.0, g) 800.0+400.0,and h) 1000.0+500.0 μM vitamin C and vitamin B_6_, respectively. Insets: (A) plot of the peak currents as a function of vitamin C concentration and (B) plot of the peak currents as a function of vitamin B_6_ concentration.

The simultaneous determination of vitamin B_6_ and vitamin C is one of the most important applications of the proposed modified electrode. This study investigated a simultaneous change in the concentrations of vitamins B6 and C by recording the DPV curves. The result showed two well-defined oxidation peaks with a 530 mV separation of the peaks (Figure 6). Insets A and B in Figure 6 show the dependence of DPV peak currents on the concentration of vitamin B_6_ and vitamin C, respectively. The sensitivities towards vitamin B_6_ in the absence and presence of vitamin C were found to be 0.0501 μA/μM (in the absence of vitamin C) and 0.0503 μA/μM (in the presence of vitamin C). These results demonstrated that the ZnFe_2_O_4_/SPGE successfully detected vitamin B6 and vitamin C simultaneously, both sensitively and selectively.

### Stability of modified electrode

For checking ZnFe_2_O_4_/SPGE sensor stability, we kept the recommended sensor within the pH equal to 7.0 in the PBS for two weeks to test ZnFe_2_O_4_/SPGE stability and, consequently, we recorded the DPV of the solution consisting of 50.0 μM vitamin B_6_ to be compared to the DPV observed prior to immersion. The oxidation peak of vitamin B_6_ did not change and, in comparison to earlier responses to the current, showed a less than 4.5 % reduction in signal, reflecting acceptable stability of ZnFe_2_O_4_/SPGE.

## Conclusion

A sensor for voltammetric determination of traces of vitamin B_6_ in real samples, based on the Zn-ferrite modified screen printed graphite electrode, was developed. The sensor exhibited a good linear response over the concentration range 0.8-585.0 μM with a detection limit of 0.17 μM for vitamin B_6_. Also, the modified electrode successfully resolves the overlapped voltammetric peaks of vitamin B_6_ and vitamin C by approximately 530 mV so that the modified electrode displays high selectivity in the DPV measurement of vitamin B_6_ and vitamin C of in their mixture solutions. As well as, the proposed method could be applied to the determination of vitamin B_6_ and vitamin C in real samples.
